# Supplementary material for: Health care providers`attitude and associated factors to safe abortion in Ethiopia, 2023: A systematic review and meta-analysis
Source: PLoS One. 2024 Sep 17;19(9):e0308419. doi: 10.1371/journal.pone.0308419 (PMC11407664; doi:10.1371/journal.pone.0308419)
Supplement: Attachment — Submitted filename: Response to Reviewres and editors, PLOSE ONE.docx [file pone.0308419.s003.docx]

**Author's response to editors and reviewers**

Health care Providers` attitude and associated factors to safe abortion in Ethiopia, 2023: A systematic review and meta-analysis.

**Authors`**

Simachew Animen Bante^1^* (SAB), Wondu Feyisa Balcha^1^ (WFB), Fentahun Alemnew Chekole^1^ (FAC), Eden Asmare Kassahun^1^ (EAK), Alemwork Abie Getu^1^ (AAG), Amlaku Mulat Awoke^1^ (AMA), and Mengistie Kassahun Tariku^2^ (MKT), Dr Endalamaw Erkie Zerihun^3^ (EEZ)

**Affiliations**

^1^Department of Midwifery, College of Medicine and Health Sciences, Bahir Dar University, Bahir Dar, Ethiopia

^2^Department of public health, College of Medicine and Health Sciences, Debre Markose University, Debre Markose, Ethiopia

^3^Department of Obstetrics and Gynecology, College of Medicine and Health Sciences, Bahir Dar University, Bahir Dar, Ethiopia

**Email addresses**

Simachew Animen Bante: [animensimachew@gmail.com](mailto:animensimachew@gmail.com)

Wondu Feyisa Balcha**:** [wondufeyisa85@gmail.com](mailto:wondufeyisa85@gmail.com)

Fentahun Alemnew Chekole: [fentahun746@gmail.com](mailto:fentahun746@gmail.com)

Eden Asmare Kassahun**:** [edenasmare86@gmail.com](mailto:edenasmare86@gmail.com)

Alemwork Abie Getu**:** [abiealemwork84@gmail.com](mailto:abiealemwork84@gmail.com)

Amlaku Mulat Awoke**:** [amlaku78@gmail.com](mailto:amlaku78@gmail.com)

Mengistie Kassahun Tariku**:** [mengesh2009@gmail.com](mailto:mengesh2009@gmail.com)

Dr Endalamaw Erkie Zerihun: [erkieendale18@gmail.com](mailto:erkieendale18@gmail.com)

***Corresponding author:** Simachew Animen Bante; [animensimachew@gmail.com](mailto:animensimachew@gmail.com)

^1^Department of Midwifery, College of Medicine and Health Sciences, Bahir Dar University, Bahir Dar, Ethiopia

**PLOS ONE**

From **Simachew Animen**

E- mail: **animensimachew@gmail.com**

**Subject: Submitting a Revised Version of the Manuscript**

**PONE-D-23-39869**

Date- 7/19/2024

**Title: - Health care Providers` attitude and associated factors to safe abortion in Ethiopia, 2023: A systematic review and meta-analysis**

We would like to thank the reviewers and editiorals for their valuable and constructive comments. We have taken advantage of their indispensable comments to improve our manuscript. In this document, we have described all changes made in accordance with the comments of reviewers. The language issue has been addressed rigorously.We believe that you will satisfy for the revision. Now, the revised manuscript with track changes and unmarked version revised paper without tracked changes are provided in the attached documents.

We are looking forward to hear from you.

With regards!

**POINT BY POINT RESPONSES**

### Dear PLOS ONE Editorial Office

Thank you for the response to the research title “**Health care Providers` attitude and associated factors to safe abortion in Ethiopia, 2023: A systematic review and meta-analysis”** Our sincerest thank also goes to reviewers for their valuable and critical remarks. We have corrected the document thoroughly according to your comments.

The point-by-point responses for each of the comments are provided in the following pages.

A. **Response to Academic Editor**
1. **Comment:** Revisions are required for the introduction, methodology, findings, and conclusion. Extensive language editing is also necessary.

**Response: Thank you! The comment is well taken. The manuscript was reviewed in detail. We invited an expert in English language and revised the documents language issue in detail as well as the grammatical errors.**

2. **Comment:** Please include the following items when submitting your revised manuscript:

- A rebuttal letter that responds to each point raised by the academic editor and reviewer(s). You should upload this letter as a separate file labeled 'Response to Reviewers'.
- A marked-up copy of your manuscript that highlights changes made to the original version. You should upload this as a separate file labeled 'Revised Manuscript with Track Changes'.
- An unmarked version of your revised paper without tracked changes. You should upload this as a separate file labeled 'Manuscript'.

**Response: Thank you! The comment is well taken. We have submitted Point by point response letter, manuscript with track changes and manuscript without track changes.**

B. **REVIEWERS’ comments response**
1. **(Reviewer 1):**
   1. **Comment:** Method: 1- Why is demographic and geographic information written in the study design section? Wouldn't it be better if these explanations were explained in the introduction?

**Response: Thank you! The comment is well taken.** **Demographic and geographic information described in the “study design “ section because this section includes not only the study design but also the study setting. This is the reason why we described here.**

2. **Comment:** Prospero code report was enough. Additional explanations in this paragraph should be deleted.

**Response: Thank you! The comment is well taken. We deleted additional explanations about Prospero.**

3. **Comment:** In the results measurement section, two variables or two results are written? (It is unclear) only one result is explained.

**Response: Thank you! The comment is well taken. We corrected in the document accordingly.**

4. **Comment:** Discussion: It has not been strongly discussed. Only Ethiopia's cultural problems and education are mentioned. Discussion requires stronger explanations to explain the results.

**Response: Thank you! The comments are well taken and we included all comments in the “discussions” section of our manuscript**

5. **Comment:** Conclusion: I think the conclusion is good and practical.

It is better to enter the number of articles of each database in the Prisma flowchart. Not just the total number

**Response: Thank you! The comment is well taken and we corrected this comment in the Fig 1, PRISMA flow chart.**

6. **Comment:** The table related to the information of the final 8 articles was not in the manuscript and the supplement file

**Response: Thank you! The comment is well taken. It is described in the result section of “characteristics of included investigations”.**
